# Risk of age older than 65 years for 30-day cardiac complication may be comparable to low-to-moderate risk according to revised cardiac risk index in non-cardiac surgery

**DOI:** 10.1038/s41598-023-42460-z

**Published:** 2023-09-20

**Authors:** Ah Ran Oh, Jungchan Park, Jong-Hwan Lee, Dahye Cha, Dan-Cheong Choi, Kwangmo Yang, Joonghyun Ahn, Ji Dong Sung, I. Hyun Park, Seung-Hwa Lee

**Affiliations:** 1 Department of Anesthesiology and Pain Medicine, Samsung Medical Center, Sungkyunkwan University School of Medicine, Seoul, Korea; 2 Department of Anesthesiology and Pain Medicine, Kangwon National University Hospital, Chuncheon, Korea; 3 Department of Biomedical Sciences, Ajou University Graduate School of Medicine, Suwon, Korea; 4 Center for Health Promotion, Samsung Medical Center, Sungkyunkwan University School of Medicine, Seoul, Korea; 5 Statistics and Data Center, Research Institute for Future Medicine, Samsung Medical Center, Seoul, Korea; 6 Rehabilitation and Prevention Center, Heart Vascular Stroke Institute, Samsung Medical Center, Sungkyunkwan University School of Medicine, Seoul, Korea; 7 Wiltse Memorial Hospital, 437, Gyeongsu-daero, Paldal-gu, Suwon-si, Gyeonggi-do Republic of Korea

**Keywords:** Cardiology, Health care, Risk factors

## Abstract

Revised cardiac risk index (RCRI) is widely used for surgical patients without containing age as a risk factor. We investigated age older than 65 years with respect to low-to-moderate risk of RCRI. From January 2011 to June 2019, a total of 203,787 consecutive adult patients underwent non-cardiac surgery at our institution. After excluding high-risk patients defined as RCRI score > 2, we stratified the patients into four groups according to RCRI and age (A: age < 65 with RCRI < 2, [*n* = 148,288], B: age ≥ 65 with RCRI < 2, [*n* = 42,841], C: age < 65 with RCRI = 2, [*n* = 5,271], and D: age ≥ 65 with RCRI = 2, [*n* = 5,698]). Incidence of major cardiac complication defined as a composite of cardiac death, cardiac arrest and myocardial infarction was compared. After excluding 1,689 patients with high risk (defined as RCRI score > 2), 202,098 patients were enrolled. The incidence with 95% confidence interval of major cardiac complication for A, B, C, and D groups was 0.3% (0.2–0.3), 1.1% (1.0–1.2), 1.8% (1.6–1.8), and 3.1% (2.6–3.6), respectively. In a direct comparison between B and C groups, old patients with RCRI < 2 showed a significantly lower risk compared to younger patients with RCRI = 2 (odd ratio, 0.62; 95% confidence interval, 0.50–0.78; *p* < 0.001). In non-cardiac surgery, the risk of age older than 65 years was shown to be comparable with low-to-moderate risk according to RCRI.

## Introduction

Age undoubtedly is a patient factor closely related to outcome of any disease. Although age is not modifiable, it is important to understand its impact on disease risk. Recently, surgical treatment tends to be more actively considered in older patients, increasing risk of negative postoperative outcome^1,2^. In non-cardiac surgery, postoperative monitoring for cardiovascular complication has been recommended for patients older than 65 years as an expert opinion^3^, but the degree of increased risk upon exceeding this age threshold has not been extensively investigated.

Revised cardiac risk index (RCRI) is a simple tool to estimate cardiac burden of patients based on six variables^4^. This index has been validated in patients undergoing surgical procedures and is now widely used for preoperative evaluation but does not include age as a variable. In this study, we aimed to evaluate cardiovascular risk of age older than 65 years in patients undergoing non-cardiac surgery with respect to RCRI. We used a large real-world cohort and enrolled patients who had low-to-moderate risk according to RCRI. These patients were stratified into four groups based on age (< 65 years vs. ≥ 65 years) and RCRI score (0 to 1 vs. 2) to analyze the effects of these two variables separately. We compared the incidence of major cardiac complications defined as a composite of cardiac death, cardiac arrest and myocardial infarction which was the outcome of interest when developing RCRI^5^ and investigated whether low-risk patients with old age show higher incidence of cardiac complications.

## Methods

The approval for this retrospective study was waived by the Institutional Review Board of Samsung Medical Center (SMC 2021–06-078) because the entire dataset for this study was extracted in de-identified form. Since data were evaluated retrospectively, pseudonymously and were solely obtained for treatment purposes, a requirement of informed consent was waived by the Institutional Review Board of Samsung Medical Center (SMC 2021–06-078). The experimental protocol was approved by the institutional committee. The present study was conducted according to the Declaration of Helsinki and was reported following the Strengthening the Reporting of Observational Studies in Epidemiology guidelines.

### Data curation and study population

To extract data from our institutional electronic archive system, we used ‘Clinical Data Warehouse Darwin-C’ of Samsung Medical Center. This is an electronic system designed to allow researchers to collect anonymous medical information from electronic hospital records. Our system contains data of more than four million patients with more than 900 million laboratory findings and 200 million prescriptions. Patient mortality outside our institution was consistently updated and verified with the National Population Registry of the Korea National Statistical Office. For this study, the Samsung Medical Center-Non Cardiac Operation (SMC-NoCop) registry (KCT 0,006,363) was used to identify participants. The SMC-NoCop is a large, single-center, de-identified cohort that consists of 203,787 consecutive adult patients who underwent non-cardiac surgery at Samsung Medical Center, Seoul, Korea, between January 2011 and June 2019. After excluding the patients with RCRI > 2 from the registry, we stratified the patients into four groups according to RCRI and age (A: age < 65 with RCRI < 2, B: age ≥ 65 with RCRI < 2, C: age < 65 with RCRI = 2, and D: age ≥ 65 with RCRI = 2).

### Study variables, definitions, and endpoints

Automatically extracted electronic medical records were used to organize preoperative and intraoperative variables by independent investigators. Comorbidity data from preoperative evaluation and from the International Classification of Diseases-10 codes were used to calculate both the RCRI and the Charlson Comorbidity Index^6,7^.

To calculate RCRI for each patient, we used the following six variables: (1) high-risk type of surgery (intrathoracic, intraperitoneal, or suprainguinal vascular surgery), (2) history of ischemic heart disease, (3) history of congestive heart failure, (4) history of cerebrovascular disease, (5) insulin therapy with diabetes mellitus, and (6) renal dysfunction (serum creatinine > 2.0 mg/dL); each one was assigned one point^7^. In this study, patients were classified as part of a low risk group (0 and 1 point) or moderate risk group (2 point) according to RCRI score.

The primary endpoint was major cardiac complications within 30 days after surgery^4^. Major cardiac complications were a composite of cardiac death, myocardial infarction, and cardiac arrest. Cardiac death was defined as sudden death or death secondary to myocardial infarction, arrhythmia, or heart failure^8^. Myocardial infarction was defined as cardiac troponin elevation with symptom presence or new electrocardiographic changes compatible with myocardial infarction following the Fourth Universal Definition of Myocardial Infarction^9^. A composite of major cardiac complication and all-cause death was compared between groups as secondary endpoints.

### Statistical analysis

Categorical variables were presented as number with percentage, and continuous variables were presented as mean ± standard deviation (SD) or median (interquartile). Outcomes were compared using a logistic regression model and were reported as odds ratio (OR) with 95% confidence interval (CI). We also performed subgroup analysis using the B and C groups to reveal unknown interactions between the observed association and variables. The results of the subgroup analysis are presented in a forest plot. All analyses were performed using R 4.0.2 (R Foundation for Statistical Computing, Vienna, Austria; http://www.R-project.org/). All tests were two-tailed, and p < 0.05 was considered statistically significant.

## Results

### Patient characteristics

**Figure 1:**
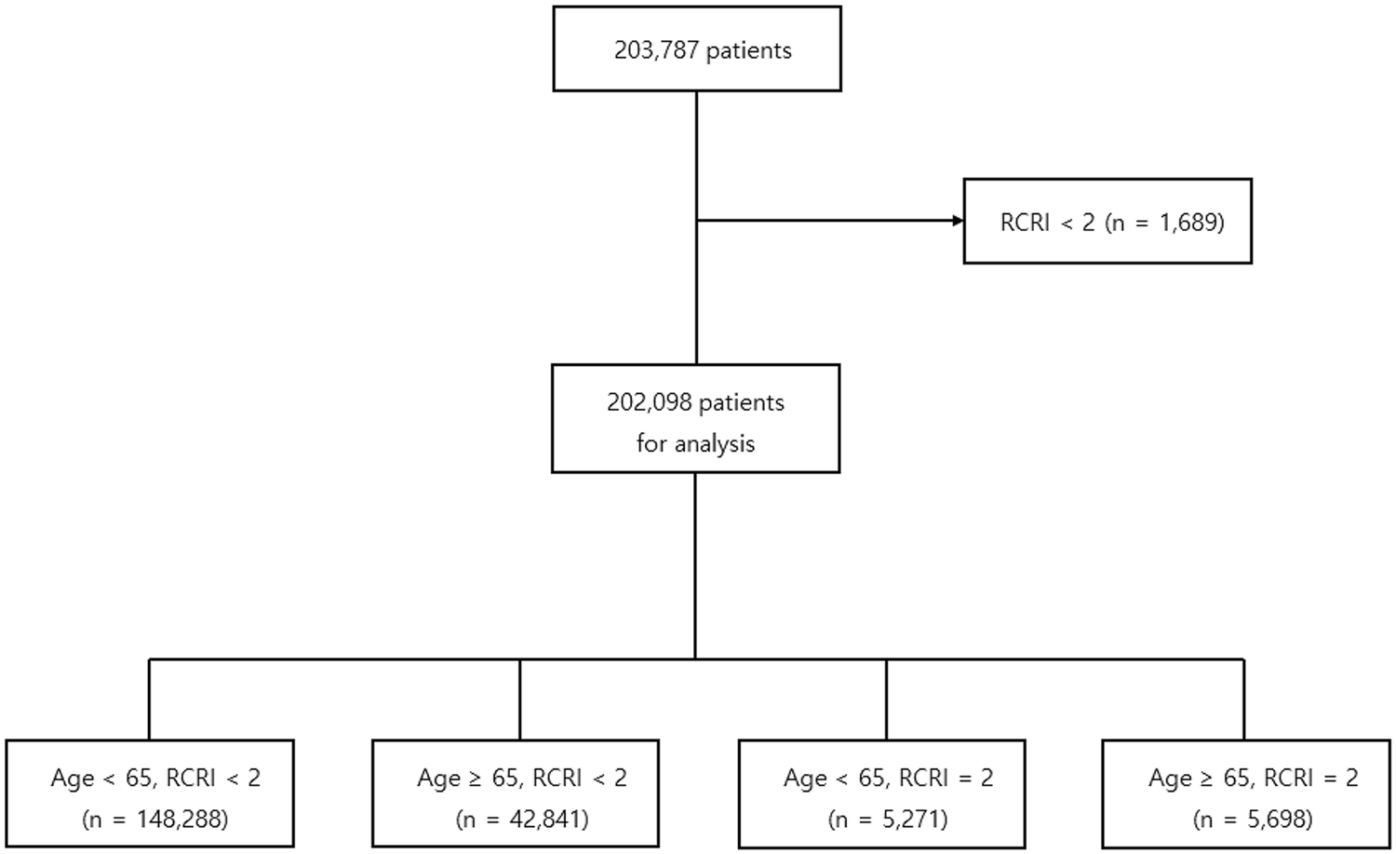
Patient flowchart.

Among 203,787 consecutive adult patients who underwent non-cardiac surgery, we excluded 1,689 patients with RCRI > 2. Thus, a total of 202,098 patients was stratified into four groups according to age and RCRI score as follows: A: age < 65 with RCRI < 2, [n = 148,288 (73.4%)], B: age ≥ 65 with RCRI < 2, [n = 42,841 (21.2%)], C: age < 65 with RCRI = 2, [n = 5,271 (2.6%)], and D: age ≥ 65 with RCRI = 2, [n = 5,698 (2.8%)] (Fig. 1). The baseline characteristics of the study participants are summarized in Table 1. Of these patients, 48,539 (24%) were older than 65 years, and 10,969 (5.4%) were RCRI = 2. Overall, there were larger numbers of comorbidities, including hypertension, diabetes, cardiovascular, and cerebrovascular disease, with higher age and RCRI score.

**Table 1:** Baseline characteristics according to revised cardiac risk index (RCRI) and age over 65 years old.

	Group A N = 148,288	Group B N = 42,841	Group C N = 5,271	Group D N = 5,698
Variables for RCRI				
High-risk surgery	38,186 (25.8)	14,754 (34.4)	4733 (89.9)	4789 (84.0)
Ischemic heart disease	480 (0.3)	929 (2.2)	518 (9.8)	1050 (18.4)
Congestive heart failure	78 (0.1)	125 (0.3)	76 (1.4)	143 (2.5)
Cerebrovasular disease	1011 (0.7)	1051 (2.5)	584 (11.1)	1027 (18.0)
Insulin treatment	2988 (2.0)	2689 (6.3)	3548 (67.3)	3957 (69.4)
Creatinine > 2 mg/dl	606 (0.4)	378 (0.9)	1083 (20.5)	430 (7.5)
Age	46.3 (± 11.9)	71.9 (± 12.7)	54.7 (± 8.6)	72.5 (± 5.4)
Male	57,880 (39.0)	21,520 (50.2)	3756 (71.3)	3746 (65.7)
Hypertension	22,223 (15.0)	21,814 (50.9)	2710 (51.4)	3794 (66.6)
Diabetes	7642 (5.2)	6705 (15.7)	3668 (69.6)	4135 (72.6)
Current alcohol	33,438 (22.5)	5344 (12.5)	920 (17.5)	707 (12.4)
Current smoking	12,688 (8.6)	1892 (4.4)	610 (11.6)	309 (5.4)
Chronic kidney disease	785 (0.5)	972 (2.3)	625 (11.9)	483 (8.5)
Previous disease				
Charlson comorbidity index	0 (0–0)	0 (0–0)	1 (0–2)	0 (0–2)
Arrhythmia	1054 (0.7)	1392 (3.2)	92 (1.7)	324 (5.7)
Peripheral artery disease	183 (0.1)	218 (0.5)	29 (0.6)	80 (1.4)
Aortic disease	158 (0.1)	349 (0.8)	29 (0.6)	127 (2.2)
Valvular heart disease	162 (0.1)	118 (0.3)	15 (0.3)	18 (0.3)
Chronic obstructive pulmonary disease	1130 (0.8)	2006 (4.7)	98 (1.9)	314 (5.5)
Operative variables				
General anesthesia	131,152 (88.4)	33,513 (78.2)	4991 (94.1)	5351 (93.9)
Emergency operation	10,422 (7.0)	2704 (6.3)	621 (11.8)	483 (8.5)
Operation duration, min	127 (± 100)	135 (± 99)	190 (± 120)	166 (± 99)
Surgery type				
Neuroendocrine	12,032 (8.1)	1002 (2.3)	27 (0.5)	9 (0.2)
Lung	6952 (4.7)	3525 (8.2)	566 (10.7)	886 (15.5)
Head & Neck	25,752 (17.4)	4841 (11.3)	191 (3.6)	222 (3.9)
Breast	16,318 (11.0)	1320 (3.1)	14 (0.3)	14 (0.20
Stomach	7724 (5.2)	2814 (6.6)	929 (17.6)	926 (16.3)
Hepatobiliary	11,208 (7.6)	3114 (7.3)	1318 (25.0)	1294 (22.7)
Colorectal	8216 (5.5)	3659 (8.5)	780 (14.8)	1053 (18.5)
Urology	10,578 (7.1)	6742 (15.7)	744 (14.1)	271 (4.8)
Gynecology	23,216 (15.7)	1253 (2.9)	26 (0.5)	32 (0.6)
Bone & Skin etc	26,292 (17.7)	14,571 (34.0)	676 (12.8)	991 (17.4)

A: age < 65 with RCRI < 2, B: age ≥ 65 with RCRI < 2, C: age < 65 with RCRI = 2, and D: age ≥ 65 with RCRI = 2.

Data are presented as n (%), mean (± standard deviation) or median (interquartile).

*RCRI* revised cardiac risk index.

### Clinical outcomes

**Figure 2:**
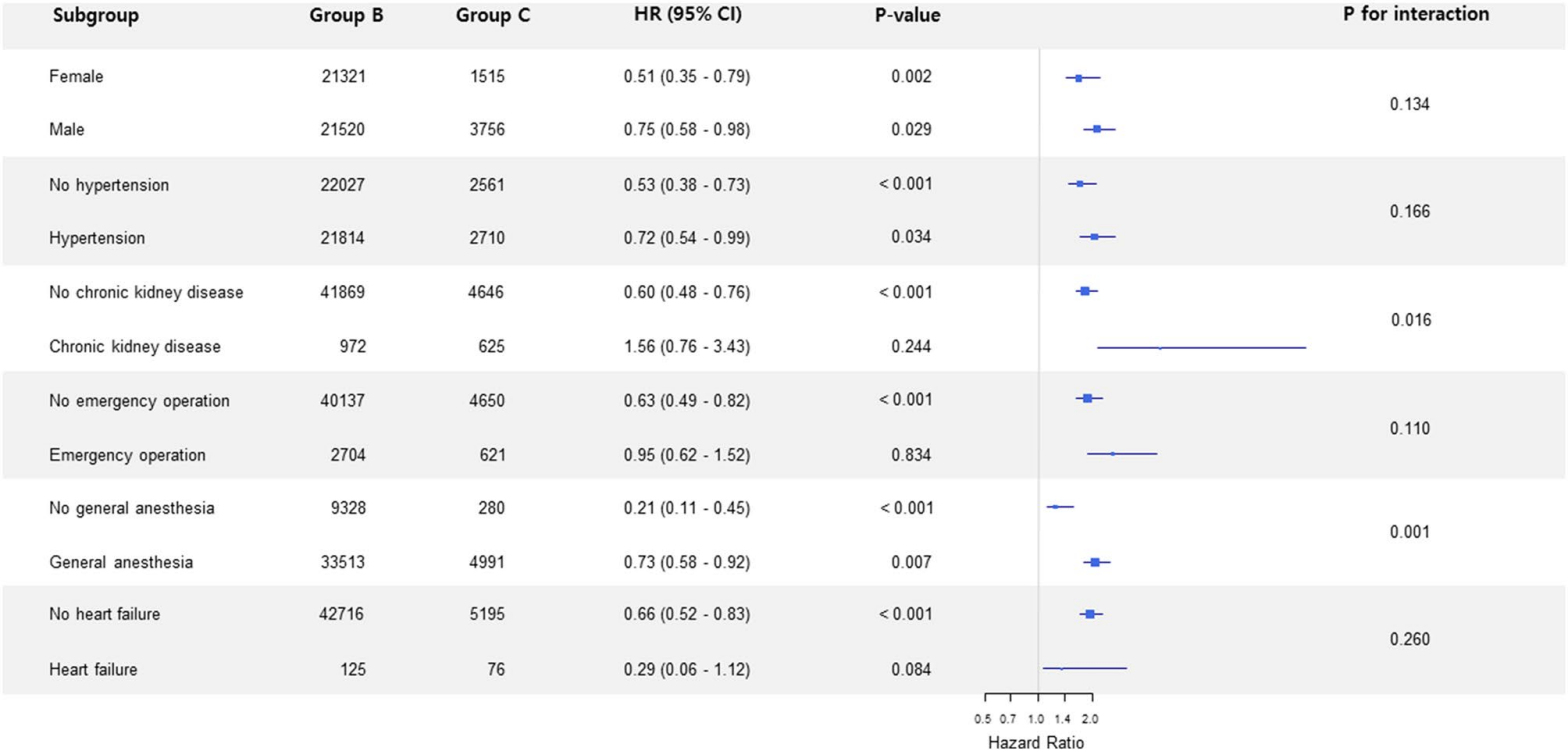
A Forest plot of subgroup analysis according to perioperative variables.

A total of 1,721 (0.85%) patients experienced major cardiac complications within 30 days after surgery. The incidence of the primary outcome with 95% CI for A, B, C, and D groups was 0.3% (0.2–0.3), 1.1% (1.0–1.2), 1.8% (1.6–1.8), and 3.1% (2.6–3.6), respectively (Table 2). Similar to the primary outcome, secondary outcomes with all-cause death also increased progressively, with increases of both age and RCRI score. We directly compared the risk of outcomes between old patients with low risk and young patients with intermediate risk (B group vs. C group). Group B showed a significantly lower risk of primary outcome compared to group C (unadjusted OR, 0.62; 95% CI, 0.50–0.78; *p* < 0.001) (Table 3). The increase of risk was also shown for all-cause death (unadjusted OR, 0.30; 95% CI, 0.24–0.39; *p* < 0.001) and a composite of major cardiac complications and all-cause death (unadjusted OR, 0.48; 95% CI, 0.40–0.57; *p* < 0.001), respectively. Subgroup analysis revealed that this association was not significant in patients with chronic kidney disease (CKD) (Fig. 2).

**Table 2:** Cardiac outcomes during 30 days follow-up according to revised cardiac risk index (RCRI) and age over 65 years old.

	Group A N = 148,288	Group B N = 42,841	Group C N = 5,271	Group D N = 5,698
Major cardiac complications	461 (0.3)	485 (1.1)	95 (1.8)	175 (3.1)
Cardiac death	38 (0.0)	36 (0.1)	13 (0.2)	16 (0.3)
Myocardial infarction	361 (0.2)	407 (1.0)	66 (1.3)	150 (2.6)
Cardiac arrest	75 (0.1)	56 (0.1)	18 (0.3)	12 (0.2)
Major cardiac complications or All-cause death	692 (0.5)	636 (1.5)	161 (3.1)	232 (4.1)
All-cause death	308 (0.2)	215 (0.5)	86 (1.6)	80 (1.4)

A: age < 65 with RCRI < 2, B: age ≥ 65 with RCRI < 2, C: age < 65 with RCRI = 2, and D: age ≥ 65 with RCRI = 2.

Data are presented as n (%).

*RCRI* revised cardiac risk index.

**Table 3:** Cardiac outcomes of age ≥ 65 with RCRI < 2 vs. age < 65 with RCRI.

Entire population	Group B N = 42,841	Group C N = 5,271	Unadjusted HR (95% CI)	*P* value
Major cardiac complications	485 (1.1)	95 (1.8)	0.62 (0.50–0.78)	< 0.001
Cardiac death	36 (0.1)	13 (0.2)		
Myocardial infarction	407 (1.0)	66 (1.3)		
Cardiac arrest	56 (0.1)	18 (0.3)		
Major cardiac complications or All-cause death	636 (1.5)	161 (3.1)	0.48 (0.40–0.57)	< 0.001
All-cause death	215 (0.5)	86 (1.6)	0.30 (0.24–0.39)	< 0.001

B: age ≥ 65 with RCRI < 2, C: age < 65 with RCRI = 2.

Data are presented as n (%).

*RCRI* revised cardiac risk index.

## Discussion

This study showed that the incidence of adverse outcomes in low-risk patients undergoing non-cardiac surgery was increased in those older than 65 years, as well as by an increase of risk to the moderate level according to RCRI score. In a comparison between old patients with RCRI < 2 and young patients with RCRI = 2, old patients with low risk showed a significantly lower incidence of adverse events than young patients with moderate risk, suggesting that the cardiovascular risk of those aged older than 65 years in non-cardiac surgery is comparable to that of low-to-moderate RCRI classification.

Major non-cardiac surgery is closely related to cardiovascular morbidity and mortality^10,11^. Elderly patients are at higher risk of these complications with reduced compliance of cardiovascular beds and complex comorbidity^12^. According to recent guidelines in non-cardiac surgery, patients older than 65 years were identified as a high-risk group that requires preoperative screening^3,13^. However, the effects of this age on cardiovascular risk has not been quantified. In this study, we divided patients by age at 65 years and compared the outcomes to evaluate risk with respect to RCRI, which is one of the most relevant cardiac risk estimators. We also compared the risk of major cardiac complications which were used as the outcome to develop RCRI^5^. In fact, exclusion of age among predicting variable remains a potential limitation, and several previous studies demonstrated an improvement of prognostic value of RCRI with an adjustment for age^14^. However, these studies were limited to specific surgery types, and the effect of including age as a variable for predictive power of RCRI remains unclear.

The primary finding of this study is that age greater than 65 years was linked to the higher incidence of major cardiac complications or all-cause death in low-to-moderate risk groups according to RCRI. Overall, the incidence of adverse cardiac complications according to RCRI stratification were similar to those of previously reported studies, suggesting that our cohort and settings well-reflected cardiac risk during the perioperative period^4^. This increase of adverse outcomes with old age was more pronounced in patients with lower RCRI score (0.5% vs. 1.5% in patients with RCRI < 2 and 3.1% vs. 4.1% in patients with RCRI = 2). This is also in line with results of previous studies reporting that the RCRI score system had greater uncertainty for those with a score of 0 to 1^4,15^. Our finding suggests that age needs to be considered as a risk factor, especially in patients with RCRI < 2, and this might resolve the uncertainty of RCRI in low-risk patients. This finding is in line with a current recommendation to screen for cardiac troponin in patients aged older than 65 years undergoing non-cardiac surgery^13^.

We also conducted a direct comparison between old patients with RCRI < 2 and younger patients with RCRI = 2. The incidence of major cardiac complications was significantly higher in patients with moderate risk according RCRI score despite younger age. This result was consistently found for a composite outcome of major cardiac complications or/and all-cause death, indicating that RCRI score well reflects cardiovascular risk after non-cardiac surgery without adjusting for age. However, our subgroup analysis revealed that this association was not significant in patients with chronic kidney disease. This might be related to increased risk of cardiovascular events in patients with chronic kidney disease. Chronic kidney disease causes a systemic and chronic proinflammatory state contributing to myocardial remodeling processes resulting in myocardial fibrosis and calcification of cardiac valves^16–18^, and these burdens from chronic kidney disease might outweigh other risks.

In this study, cardiac death accounted for about 15% (103/689) of all death, and RCRI score also showed an association with all-cause mortality. Although the design of RCRI was initially focused on cardiac-related complications, subsequent studies have made efforts to expand its application to all-cause mortality. Several previous studies demonstrated a proportional relationship between higher RCRI and mortality in non-cardiac surgery^19–22^, but a systematic investigation reported low performance and diagnostic accuracy of RCRI for predicting all-cause mortality^4^. However, evidence has been continuously accumulating on the effectiveness of RCRI for predicting all-cause mortality^23–25^, and addition of new variables to improve the predictive power has been attempted^23–25^. These cumulative investigations suggest that the RCRI can be used for risk predictions of all-cause mortality beyond adverse cardiac events. Our results present a possibility that retaining an age factor in calculation of RCRI score may improve the performance for all-cause mortality, but this requires further investigations.

There are several noteworthy limitations when interpreting our results. First, there is a possibility of selection bias because we excluded patients at the highest risk of cardiovascular outcomes as defined by RCRI. As this might have resulted in an underestimation of cardiovascular events, our results should be verified against the full spectrum of RCRI risk scores for generalization. Second, perioperative management has evolved over time but was not considered in our analysis. In addition, perioperative managements may vary between the departments. This methodological limitation might have led to an under- or overestimation of cardiovascular risk. Third, routine tests of postoperative electrocardiogram and cardiac enzyme were not performed in all patients, which might have affected the detection of cardiovascular events. Especially for myocardial infarction, an evaluation such as coronary angiogram was at the discretion of cardiologists. Despite these limitations, our study is notable in that we evaluated postoperative cardiovascular risk of age older than 65 years in respect to RCRI. Our findings should encourage more active screening for cardiovascular complications in patients aged older than 65 years, as suggested by recent guidelines in non-cardiac surgery.

## Conclusion

In patients with low-to-intermediate risk according to RCRI, age older than 65 years showed higher incidence of major cardiac complications or all-cause death after non-cardiac surgery. Further prospective studies on the effect of age as a cardiovascular risk factor of non-cardiac surgery are warranted to confirm our findings.

## Data Availability

The datasets generated during and/or analysed during the current study are available from the corresponding author on reasonable request.
